# Enhancing Lymphoma Diagnosis, Treatment, and Follow-Up Using ^18^F-FDG PET/CT Imaging: Contribution of Artificial Intelligence and Radiomics Analysis

**DOI:** 10.3390/cancers16203511

**Published:** 2024-10-17

**Authors:** Setareh Hasanabadi, Seyed Mahmud Reza Aghamiri, Ahmad Ali Abin, Hamid Abdollahi, Hossein Arabi, Habib Zaidi

**Affiliations:** 1 Department of Medical Radiation Engineering, Shahid Beheshti University, Tehran 1983969411, Iran; setareh.hasanabadi@gmail.com (S.H.); smr-aghamiri@sbu.ac.ir (S.M.R.A.); 2 Faculty of Computer Science and Engineering, Shahid Beheshti University, Tehran 1983969411, Iran; a_abin@sbu.ac.ir; 3 Department of Radiology, University of British Columbia, Vancouver, BC V5Z 1M9, Canada; habdollahi@bccrc.ca; 4 Department of Integrative Oncology, BC Cancer Research Institute, Vancouver, BC V5Z 1L3, Canada; 5 Division of Nuclear Medicine and Molecular Imaging, Geneva University Hospital, CH-1211 Geneva, Switzerland; hossein.arabi@unige.ch; 6 Department of Nuclear Medicine and Molecular Imaging, University of Groningen, University Medical Center Groningen, 9700 RB Groningen, The Netherlands; 7 Department of Nuclear Medicine, University of Southern Denmark, 500 Odense, Denmark; 8 University Research and Innovation Center, Óbuda University, 1034 Budapest, Hungary

**Keywords:** lymphoma, ^18^F-FDG PET/CT, radiomics, genomics, radiogenomics, deep learning, personalized therapy

## Abstract

**Simple Summary:**

Lymphoma is a type of cancer that affects the immune system and can be difficult to diagnose and treat effectively, especially in the early stages. Current imaging methods, such as PET/CT scans, are valuable tools for diagnosing and monitoring the disease, but they have limitations in providing precise information for personalized treatment plans. Recently, radiomics and artificial intelligence (AI) have emerged as promising technologies that can analyze detailed patterns in medical images, helping to uncover information that might not be visible to the human eye. This study explores the potential of these technologies in improving the diagnosis, staging, and treatment selection for lymphoma patients. However, further research is needed to confirm their reliability and ensure they can be effectively used in clinical practice.

**Abstract:**

Lymphoma, encompassing a wide spectrum of immune system malignancies, presents significant complexities in its early detection, management, and prognosis assessment since it can mimic post-infectious/inflammatory diseases. The heterogeneous nature of lymphoma makes it challenging to definitively pinpoint valuable biomarkers for predicting tumor biology and selecting the most effective treatment strategies. Although molecular imaging modalities, such as positron emission tomography/computed tomography (PET/CT), specifically ^18^F-FDG PET/CT, hold significant importance in the diagnosis of lymphoma, prognostication, and assessment of treatment response, they still face significant challenges. Over the past few years, radiomics and artificial intelligence (AI) have surfaced as valuable tools for detecting subtle features within medical images that may not be easily discerned by visual assessment. The rapid expansion of AI and its application in medicine/radiomics is opening up new opportunities in the nuclear medicine field. Radiomics and AI capabilities seem to hold promise across various clinical scenarios related to lymphoma. Nevertheless, the need for more extensive prospective trials is evident to substantiate their reliability and standardize their applications. This review aims to provide a comprehensive perspective on the current literature regarding the application of AI and radiomics applied/extracted on/from ^18^F-FDG PET/CT in the management of lymphoma patients.

## 1. Introduction

A variety of diseases that result from the clonal proliferation of lymphocytes are known as malignant lymphomas [1]. These conditions can be classified into two primary types: Hodgkin lymphoma (HL) and non-Hodgkin lymphoma (NHL). HL comprises a relatively small portion, accounting for approximately 10% of all lymphoma cases, while NHL constitutes the majority, representing roughly 90% of all lymphoma cases. While T cells, or natural killer cells, can also be responsible for lymphomas, the majority (90%) originate from B cells [2]. In 2023, NHL is expected to account for 4.1% of new cancer diagnoses and 3.3% of cancer-related deaths in the United States of America, while HL is projected to represent 0.5% of new diagnoses and 0.1% of deaths during the same period [3,4].

Positron emission tomography combined with computed tomography using [^18^F]-fluorodeoxyglucose (^18^F-FDG PET/CT) is widely employed as a noninvasive three-dimensional imaging modality in the management of lymphoma patients. ^18^F-FDG PET/CT has different indications in lymphoma patients in various aspects, including initial staging before treatment, re-staging, evaluation after treatment, monitoring therapy progress, follow-up post-therapy, and assessing disease transformation. However, it is important to acknowledge that this technology has limitations, including variations in FDG avidity among various lymphoma subtypes, as well as the potential for false-negative and false-positive results [5,6].

Radiomics is a rapidly advancing research field focused on quantitatively extracting features from medical images and transforming them into rich and high-dimensional data. The AI learning process begins with data collection and preprocessing, followed by feature extraction, model training, and evaluation. The analysis of these data provides unique biological insights, enhancing our understanding of disease processes and supporting clinical decision-making [7,8,9]. Furthermore, ^18^F-FDG PET/CT radiomics enhances diagnosis, facilitates the development of individualized treatment strategies, and refines outcome predictions based on factors like tumor heterogeneity and lymphoma’s biological, pathological, and metabolic characteristics. This non-invasive technique has gained significant attention in the clinical setting owing to its versatile applications [10,11].

In recent years, the integration of artificial intelligence (AI) into the field of medical imaging has had a profound impact on patient care, disease detection, diagnosis, and treatment planning [12,13,14,15]. AI algorithms enable the segmentation of medical images, automated analysis, disease identification, risk assessment, therapy response assessment, and prognosis prediction [16,17,18,19]. AI also optimizes image quality, allowing for quantitative analysis of imaging biomarkers, and provides clinicians with decision-support tools through the integration of various patient data [20]. Leveraging these cutting-edge methods can lead to improved lymphoma diagnosis and treatment, resulting in more successful personalized medicine strategies [21].

Despite the continuous growth of AI research in the medical field, numerous challenges require further investigation and research, particularly with respect to the availability of diverse and extensive datasets and multi-center validation studies.

The objective of this study is to evaluate how radiomics and AI tools using ^18^F-FDG PET/CT can effectively enhance the accuracy of lymphoma diagnosis, optimize treatment strategies, and improve disease monitoring. This assessment will focus on the role of AI in delivering personalized treatment options and ultimately leading to better clinical outcomes for patients with lymphoma.

## 2. Literature Search Strategy

To conduct this review, we undertook a comprehensive exploration of the PubMed database using a diverse set of search phrases and keywords. These search terms included ‘lymphoma’, ‘artificial intelligence’, machine learning’, ‘radiomics’, ‘deep learning’, and ‘radiogenomics’. Our search covered content from the year 2000 to 20 August 2024. We used the Boolean operators ‘AND’ and ‘OR’ to combine the main terms and keywords, refining the search results. Publications covering a wide range of diseases, conference papers, literature reviews, and articles in non-English languages were excluded using specific criteria to narrow down our study selection. A limitation of this study is the use of a single database (PubMed) for the bibliography search, which may have limited the inclusion of studies available in other databases. It is important to note that a limitation of this study is the use of a single database (PubMed) for the bibliography search, which may have limited the inclusion of studies available in other databases.

## 3. Literature Search Outcome

Primarily, in the first step of our research, 227 articles were selected. After rigorously applying the specified inclusion/exclusion criteria outlined in the Section 2, we meticulously identified a total of 128 articles that were considered relevant. It is important to note that the majority, amounting to up to 95% of the research, was conducted using a retrospective design. These findings, along with a detailed summary of the studies, can be found in Table 1 and Table 2.

**Figure 1:**
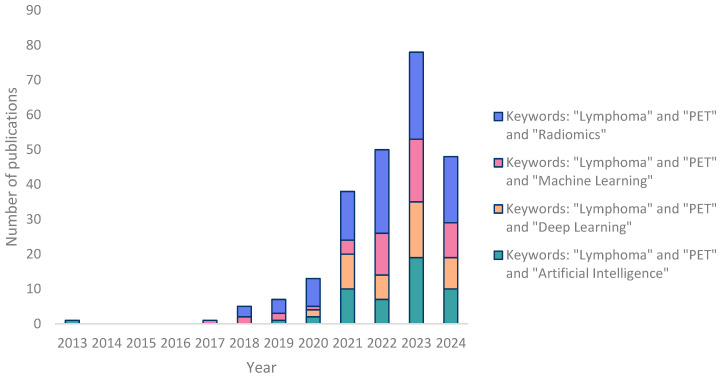
Bar chart showing the number of publications according to years (the search was conducted from the year 2000 to 20 August 2024. No articles were found on PubMed prior to 2013).

**Figure 2:**
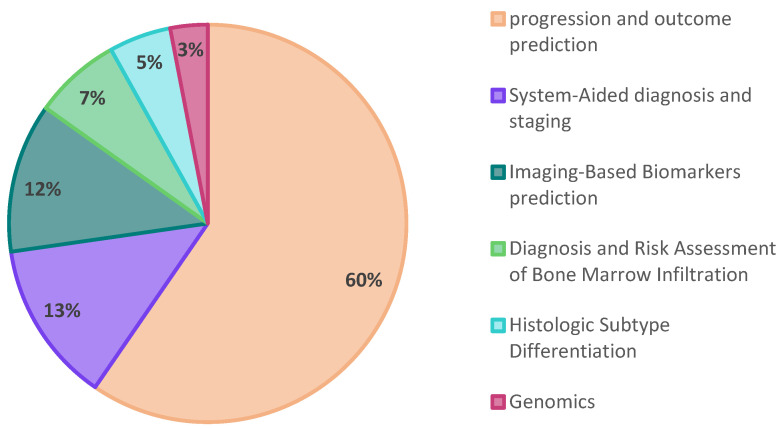
A pie chart illustrating the distribution of publications based on the study subject.

As expected, the number of studies using radiomics and/or AI has significantly risen since 2020, and the keywords ‘Lymphoma’, ‘PET’, and ‘Radiomics’ together seem to lead to more relevant results (Figure 1). In the subsequent step, we meticulously categorized the studies into five distinct sections, as visually represented in Figure 2. The majority of studies were related to the topics of “progression and outcome prediction.” In contrast, genomics studies comprised the smallest share of the research.

As a result, our findings revealed that the predominant approach in lymphoma research involves employing radiomics or a combination of radiomics with AI for disease progression and outcome prediction of patients. Notably, similar to other research in this domain, these studies employed a retrospective design.

## 4. Summary of Findings and Discussion

### 4.1. From Image to Clinical Decision: Potential of ^18^F-FDG PET/CT Radiomics in Lymphoma

^18^F-FDG PET/CT is widely utilized for lymphoma staging, diagnosis, and treatment guidance today [5]. However, it has limitations due to increased FDG uptake in organs infiltrated post-immunochemotherapy, infections, and inflammation. Furthermore, lymphoma may not consistently exhibit high uptake, which complicates therapy response assessment [22,23]. These challenges present difficulties for physicians in image interpretation and clinical decision-making.

With the growing demand for advanced diagnostic tools in lymphoma management encompassing various clinical scenarios, such as early and precise diagnosis, staging, prognosis assessment, and treatment response evaluation, the fields of radiomics and AI have increasingly come to the forefront of lymphoma research. In the upcoming sections, we will explore the potential benefits of ^18^F-FDG PET/CT radiomics and AI-powered tools in diverse clinical situations.

#### 4.1.1. Diagnosis and Risk Assessment of Bone Marrow Infiltration (BMI)

Evaluation of bone marrow infiltration in lymphomas has significant effects on prognosis and therapeutic management. The gold standard procedure for this assessment has historically been bone marrow biopsy (BMB). Concurrently, ^18^F-FDG-PET has been recognized as an important staging tool for lymphomas and may serve as a non-invasive BMB supplant or replacement. Ongoing research and debates are still being conducted on the clinical use of ^18^F-FDG-PET to assess BMI in lymphomas [24]. To identify BMI in patients with lymphoma, radiomic features extracted from ^18^F-FDG PET/CT images provide an additional tool for visual assessment [25].

**Figure 3:**
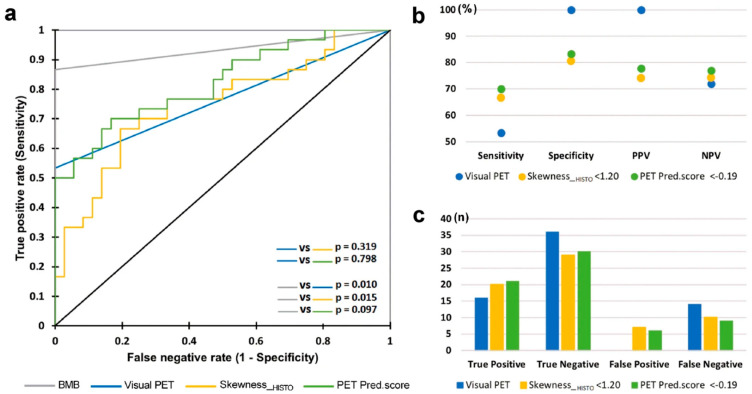
ROC curves for BMI diagnosis through BMB, visual PET, skewness_HISTO PET, and pred.score PET evaluations. (**a**) Comparative ROC curves; (**b**) evaluation of sensitivity, specificity, PPV, and NPV; (**c**) rates of true positives, true negatives, false positives, and false negatives. Reprinted with permission from [26] under a Creative Commons Attribution 4.0 International License.

In this regard, Faudemer et al. demonstrated the potential of FDG-PET/CT radiomics for diagnosing BMI in Follicular lymphoma (FL) patients [26]. Furthermore, when comparing diagnostic methods, they identified a significant difference between BMB and visual PET assessments (*p*-value = 0.010). However, there was no significant difference between BMB and PET pred.score assessments (based on radiomic features), as depicted in Figure 3. To enhance the diagnostic capabilities of FDG-PET in this context, further exploration of skeleton texture analysis is recommended.

In a comprehensive study involving 82 patients diagnosed with Diffuse Large B Cell Lymphoma (DLBCL), Aide et al. identified that the radiomics feature SkewnessH, a first-order metric, demonstrated a sensitivity of 81.8% and specificity of 81.7% in accurately detecting BMI in baseline FDG PET/CT [27]. This study underscored the pivotal role of skeletal textural characteristics in BMI diagnosis and the improvement of risk assessment for DLBCL patients. Conversely, in another study involving DLBCL patients, no significant differences were observed in textural features extracted from the femurs and iliac regions between patients with positive and negative BMB patients [28]. However, two parameters derived from the grey-level zone-length matrix in the iliac crests exhibited a tendency for higher values in the BMB-positive group compared to the BMB-negative group. It is important to note that these differences did not reach statistical significance after applying multiple comparison corrections. The limited number of patients in the study may have influenced the research outcomes. Incorporating larger sample sizes and conducting repeated studies will enhance the accuracy and reliability. In an effort to demonstrate the value of FDG-PET radiomics in predicting BMI in mantel cell lymphoma (MCL) patients, it has been found that FDG-PET texture features significantly enhanced SUV-based prediction (AUC 0.73 vs. 0.66). Furthermore, combining these radiomic features with laboratory data can further enhance the predictive performance (AUC = 0.81) [29].

**Figure 4:**
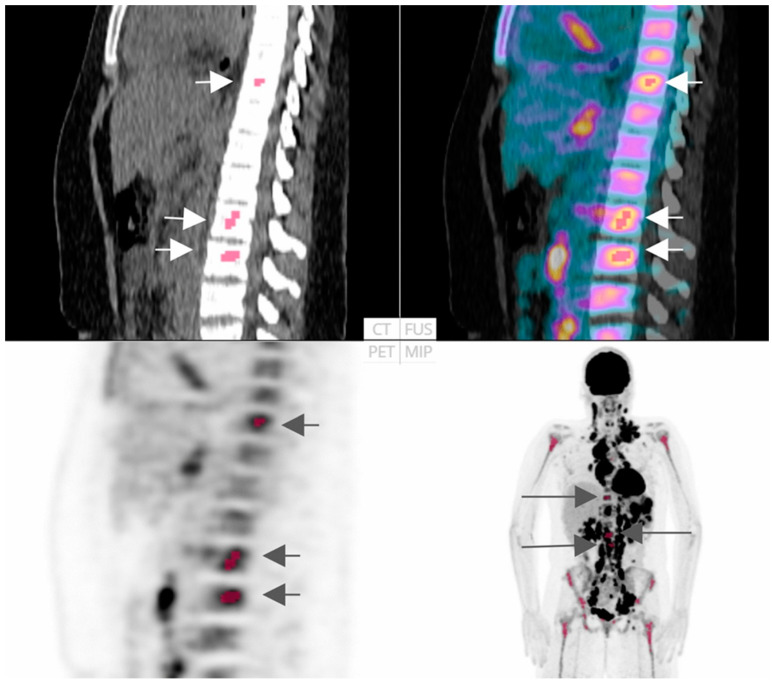
FDG-PET/CT representation: An instance where both AI-driven technique and the majority (8 out of 10) of physicians identified localized uptake in the skeletal/bone marrow (indicated in red and with arrows). Reprinted with permission from [30] under a Creative Commons Attribution 4.0 International License.

In another remarkable study, Sadik et al. proposed an innovative AI-based approach aimed at detecting bone marrow uptake (BMU) in HL patients [30]. Their findings revealed an impressive concordance rate (81%) between the AI’s evaluation of BMI and assessments by physicians. Furthermore, AI quantified diffuse BMU, showcasing its potential for clinical application and demonstrating performance comparable to the manual method, as illustrated in Figure 4. Similarly, in a related study, employing AI to highlight suspicious BMU in HL patients significantly enhanced interobserver agreement among physicians from various hospitals [31].

Despite the prognostic value of FDG-PET/CT radiomics in accurately detecting BMI and enhancing risk assessment in lymphoma patients, only a limited number of studies have explored its full potential up to now [11,25,26,27,28,29,30,31]. Due to the importance of BMI in lymphoma patients, especially B-cell lymphoma [B-cell lymphoblastic lymphoma 40–60%, small lymphocytic lymphoma 85%, MCL 50–80%, FL 40–70%], and in Aggressive NK cell leukemia (>95%) and also in Lymphocyte depleted (>50%) [32], it is necessary to conduct further research in the field. Future research can focus on various subtypes of lymphoma or larger and diverse datasets and explore its clinical utility.

#### 4.1.2. Histologic Subtype Differentiation: Radiomics and Future Directions

The current gold standard for diagnosing most hematopoietic and lymphoid tissue malignancies still involves the pathological examination of the afflicted tissue, typically obtained through surgical resection [33]. There are several pitfalls associated with this approach, including errors arising from insufficient data/tissue acquisition, personal subjectivity, and complexities in the classification and diagnosis of lymphomas, as well as errors related to molecular genetic tests [34]. Moreover, the invasive nature of this method, along with the fact that it may not adequately capture relapsed or refractory cases, can significantly impact treatment decisions.

Different subtypes of bulky mediastinal lymphomas, such as classical HL (CHL), gray zone lymphoma, and primary mediastinal B-cell lymphoma (PMBCL), exhibit distinct FDG-PET characteristics and metabolic heterogeneity. Machine learning (ML) combined with radiomics can help in creating a visual representation of these masses to aid in the histological differentiation of mediastinal bulky lymphomas [35].

In a retrospective investigation carried out by de Jesus et al. involving 120 patients, it was found that a radiomics-driven model exhibited superior performance compared to SUV_max_-based models when it came to discriminating between FL and DLBCL (area under the curve (AUC) of 0.86 vs. 0.79, accuracy of 80% vs. 0.70) [36]. Furthermore, Lovinfosse et al. reported a moderate level of agreement among observers in the diagnosis of HL versus DLBCL [37]. The best ML models for differentiating DLBCL from HL showed promising results (AUC of 0.95 in the lesion-based approach using TLR (tumor-to-liver ratio) radiomics, and an AUC of 0.86 in the patient-based approach utilizing original radiomics and age).

Yang et al. successfully developed DL-CAD systems based on PET/CT images to address the clinical challenge of differentiating histological components in enlarged cervical lymph nodes, particularly between lymph node metastasis and lymphoma involvement [38]. Their findings demonstrated that combining handcrafted and DL-based features improved diagnostic performance. The integration of these systems in clinical practice holds promise for improving therapeutic intervention quality and optimizing patient outcomes. Recent research emphasized the usefulness and potential of radiomic features and machine learning-based radiomics/deep learning models in distinguishing between different tumor types, such as primary CNS lymphoma (PCNLS) and glioblastoma [39], breast DLBCL and breast invasive ductal carcinoma (IDC) [40], breast carcinoma from breast lymphoma [41], pancreatic carcinoma from mass-forming pancreatic lymphoma [42], differentiation between thymic epithelial tumors (TETs) and mediastinal lymphomas (MLs) [43], lymphoma from liver and lung cancers [44], sarcoidosis and malignant lymphoma [45], renal cell carcinoma and renal lymphoma [46], squamous cell carcinoma and NHL in the oropharynx [47], and in enhancing the diagnostic performance for PCNLS and brain metastases [48], and sarcoidosis and lymphoma [37,45]. A limited number of studies focused on distinguishing the histological subtypes of lymphoma itself. For differentiating Diffuse Large B-Cell Lymphomas (DLBCLs) and mucosa-associated lymphoid tissue (MALT) lymphomas, Total Lesion Glycolysis (TLG) achieved an AUC of 0.906, sensitivity of 0.900, and specificity of 0.780 [49].

A recent study introduced *PST-Radiomics*, a method that enhances lymphoma classification by capturing intra-tumor metabolic heterogeneity (ITMH) using PET/CT imaging. This approach outperformed traditional methods in subtype classification by analyzing tumor segments with a specialized neural network [50].

Future studies should focus on differentiating other subtypes using multiclass classification ML and Deep Learning (DL) algorithms. Including larger datasets and external validation will enhance the reliability of this approach compared to traditional biopsy methods.

#### 4.1.3. Progression and Outcome Prediction

Currently, ^18^F-FDG PET/CT stands as the standard imaging technology for diagnosing, staging, and predicting prognosis in both HL and NHL patients. It has been widely used in managing lymphoma patients, and its importance in prognostication is steadily growing [51]. Additional insights obtained from ^18^F-FDG PET/CT scans, particularly through radiomics-based analysis to assess tumor heterogeneity, have the potential to enhance prognostic accuracy. The integration of radiomics and AI into prediction prognosis assessment is under investigation, either in combination with existing prognostic tools or as standalone markers. These advancements aim to facilitate more precise prognosis predictions and enhance clinical decision-making for patients with lymphoma. In this section, we will explore the application of AI and radiomics in lymphoma risk stratification.

Diffuse aggressive NHL in adults can achieve complete remission in a substantial percentage, ranging from 60% to 80%. However, a challenging aspect of managing this disease is that from 20% to 40% of patients may experience relapses. Similarly, HL responds well to chemotherapy, leading to potential cures for the majority, but from 5% to 10% may have refractory disease initially, and from 10% to 30% may relapse [52,53]. Despite the existence of clinical prognostic tools, like the International Prognostic Index (IPI) for risk assessment, accurate prognosis prediction remains a challenge due to the significant heterogeneity within lymphoma. NHL encompasses various subtypes, each with distinct characteristics, making it complex to identify risk factors that contribute to our understanding of underlying biological mechanisms and clinical practice [54,55].

The majority of studies conducted in the field of progression and outcome prediction have focused on DLBCL due to its widespread occurrence and significant clinical importance within the field of lymphomas [2]. A combined clinical and PET/CT radiomics model demonstrated superior performance compared to a model solely based on metabolic tumor volume (MTV) (AUC = 0.75 vs. AUC = 0.67) when it came to predicting 2-year event-free survival (2-EFS) for DLBCL patients who received R-CHOP treatment [56]. Another study suggested that integrating AI-generated imaging metrics into clinical workflows enhances risk stratification and improves central nervous system relapse prediction in DLBCL, surpassing the IPI [progression-free survival (PFS): 1.87 vs. 1.38; overall survival (OS): HR = 2.16 vs. 1.40] [57].

It is worth noting that a novel radiomics feature selection approach has effectively demonstrated lesion heterogeneity within HL patients. The findings by Sollini et al. underscore the importance of considering multiple lesions for accurate patient outcome prediction, as relying solely on the largest lesion was found to be unreliable [58].

Another research effort aimed to predict PFS in patients with CHL after salvage chemotherapy and autologous stem-cell transplant [59]. A predictive machine learning model was developed using ^18^F-FDG PET radiomics and clinical data for 113 classical Hodgkin lymphoma patients, identifying high-risk individuals with lower 3-year PFS. The model achieved AUCs of 0.810 in the training cohort and 0.750 in the validation cohort. Adding radiomic features such as MTV and TLR, SUV_mean_ improved performance, while run-length non-uniformity was an independent survival predictor in DLBCL patients [60]. Moreover, when combined with the IPI, it forms a valuable risk stratification model, guiding personalized treatment strategies.

Attempts to improve the prediction of OS and PFS in DLBCL patients using hybrid nomograms that combine the radiomics score (RS) with IPI showed promising results. It seems that the application of radiomics analysis to metabolic bulk volume (MBV) has emerged as a potential approach for assessing prognosis [61]. In a study performed on aggressive B-cell lymphoma, certain radiomic features, notably gray-level non-uniformity (GLNU) and kurtosis—measuring the “tailedness” of the intensity distribution within lymphoma tumors on ^18^F-FDG PET/CT scans; high kurtosis indicating a sharp peak and heterogeneous metabolic activity, while low kurtosis suggesting uniform activity—were correlated with OS (*p* = 0.035). Tumor texture was unable to forecast the response to treatment [62].

In another study with DLBCL patients who were treated with the RCHOP regime, PET radiomics showed promise for prognosis [63]. Radiomic features, including aRS, strongly predicted PFS, cause-specific survival (CSS), and OS in the testing and validation cohorts, with corresponding AUCs of 0.709, 0.721, 0.740, and 0.706, 0.763, and 0.703, respectively. Utilizing PET/CT radiomics, a research effort established a predictive nomogram for assessing DLBCL patients’ PFS during immunochemotherapy, incorporating RS, blood platelet levels, and gender [64]. A recent study presented a promising multimodal deep learning model for predicting treatment failure in DLBCL patients using PET/CT imaging, achieving a high accuracy (91.22%) and an AUC of 0.926 in the primary dataset while maintaining strong performance (88.64% accuracy and an AUC of 0.925) in the external dataset [65].

Employing machine learning in PET-based radiomics showed potential for predicting DLBCL treatment outcomes. This includes SUV_max_ and Gray-Level Co-Occurrence Matrix (GLCM) dissimilarity, which served as independent predictors for response to therapy [66]. In another study, involving 271 DLBCL patients, a combined model was developed to identify high-risk patients after first-line therapy. This model incorporated clinical features, baseline, end-of-therapy (EoT) PET radiomic features, and delta PET features. The study demonstrated superior prognostic performance compared to traditional models, such as the International Prognostic Index (IPI) and Deauville score (DS), offering improved risk stratification and enhanced clinical decision support [67]. In a dual-center retrospective analysis of DLBCL patients, endeavors to predict 2-EFS using radiomics and clinical data yielded promising results (AUC of 0.85) [68].

In a study conducted by Jiang et al., the authors developed a PET radiomics signature (Rad-Sig) to predict DLBCL patient survival [69]. The study’s results demonstrated a significant association between Rad-Sig and both PFS and OS. Models that integrated Rad-Sig in combination with clinical factors exhibited superior performance compared to other models (C-index of 0.801 for PFS, 0.807 for OS). Moreover, it should be emphasized that external validation confirmed the effectiveness of these models in predicting patient survival.

An additional investigation has identified the predictive potential of PET radiomic features in primary gastrointestinal DLBCL patients obtaining treatment with an R-CHOP-like regimen. These investigations have revealed that the incorporation of Rad-Sig together with metabolic and clinical variables can robustly forecast patient outcomes with greater accuracy when compared to clinical models and the IPI score, underscoring its value in facilitating personalized treatment decisions [70].

**Figure 5:**
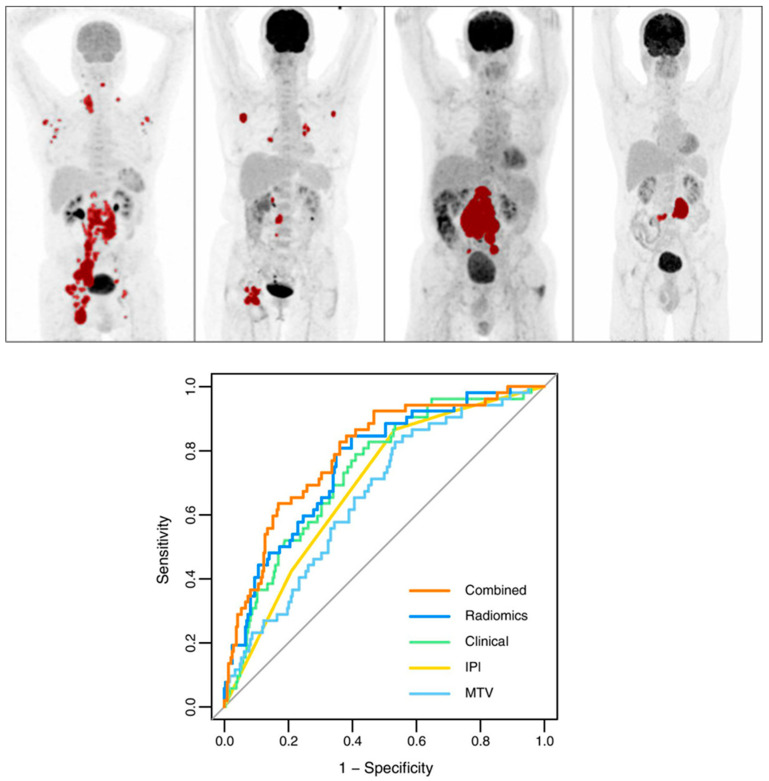
Top panel: Maximum intensity projections showcasing patients categorized by MTV and dissemination levels. Tumors are marked in red. Sequence from left to right: high MTV with high dissemination, low MTV with high dissemination, high MTV with low dissemination, and low MTV with low dissemination. Bottom panel: Receiver operating characteristic curves for the 2-year progression timeframe based on IPI, best clinical approach, MTV, the top radiomics model, and combined prediction models. Modified and reprinted with permission from [71] under a Creative Commons Attribution 4.0 International License.

Eertink et al. [71] emphasized that integrating baseline radiomics with commonly used clinical predictors significantly enhances the prediction of treatment outcomes. This combination improved model performance, resulting in a 15% increase in positive predictive value (PPV), and allowed for more precise identification of high-risk patients compared to the IPI model. The clinical predictors in this study included key factors such as WHO performance status and age above 60 years. As shown in Figure 5, the IPI model achieved an AUC of 0.68, the optimal radiomics model reached an AUC of 0.76, and the combined model, incorporating both radiomic and clinical features, achieved an AUC of 0.79.

The research conducted by Eertink and other colleagues underscores the enhanced precision in identifying high-risk patients through the utilization of baseline ^18^F-FDG-PET radiomic features, surpassing the conventional IPI risk score [72]. Furthermore, their clinical PET model, developed from the HOVON-84 dataset, consistently demonstrated its predictive efficacy across six independent studies.

**Figure 6:**
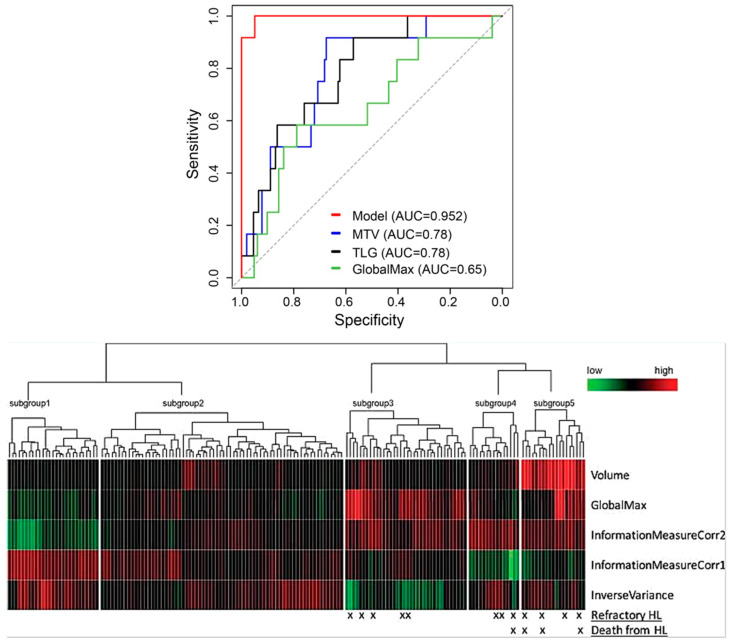
Top panel: Receiver Operating Curves showcasing the performance of models incorporating five radiomic characteristics (in red), metabolic tumor volume (in blue), total lesion glycolysis (in black), and GlobalMax (SUV_max_, represented in green) for the patient group with mediastinal conditions. Bottom panel: Heatmap illustrating the prognostic categories derived from the top five mediastinal radiomic predictors. Modified and reprinted with permission from [74] under a Creative Commons Attribution 4.0 International License.

Irrespective of the selected lesion or feature selection techniques, Eertink, in collaboration with other researchers, found that patient-level conventional PET features and dissemination features exhibited the most substantial predictive potential for DLBCL patient progression within a 2-year period (AUC range of 0.72–0.75). Nevertheless, textural and morphological radiomic features did not contribute to improving the predictive value [73]. Incorporating first- and second-order radiomic features in a PET scan model enhanced outcome prediction for early-stage Hodgkin lymphoma, as indicated in Figure 6. This approach also held promise for identifying patients with a higher risk of mortality, pointing toward personalized risk-adapted management (AUC = 95.2%) [74].

Using deep learning and rule-based reasoning, a recent study predicted treatment response in B-cell lymphomas based on pre-treatment CT and PET images, surpassing traditional methods (in the lesion-based approach on one whole slice, AUC = 0.93 for PET vs. 0.91 for diagnostic quality CT and 0.92 for low-dose CT) [75]. Another investigation focusing on DLBCL patients revealed that the utilization of maximum intensity projection (MIP) images from ^18^F-FDG PET baseline scans with a CNN model enhanced the prediction of 2-year time-to-progression (TTP) when compared to IPI (AUC of 0.74, vs. 0.68) [76].

A radiomics-based model for predicting 2-EFS in cHL patients is feasible. The most effective model in their dataset was built using ridge regression. Nevertheless, no notable distinction was observed between this model and a logistic model that included MTV and clinical features. However, further research is required to establish optimal clinical thresholds [77]. The pre-treatment ^18^F-FDG PET/CT radiomics model effectively predicted ibrutinib treatment response in diverse lymphoma patients, outperforming traditional PET metrics [78]. Combining intra-tumoral heterogeneity features, PET metrics, and clinical risk factors improves survival predictions for untreated DLBCL patients compared to using only the IPI. The prognostic model, when stratified by cell-of-origin (COO) subgroups, identified a high-risk population with lower 2-year survival rates compared to the IP [79].

Specific combinations of PET and CT radiomics with clinical parameters independently predicted patient survival and the need for radiotherapy in HL patients [80]. On the other hand, in another study involving extranodal natural killer (NK)/T cell lymphoma (ENKTL) patients, a radiomics-dependent model demonstrated lower performance when compared to the metabolism-based model. The inferior performance observed could be attributed to the retrospective study design or the small dataset size [81].

Zhao et al. demonstrated that the integration of ^18^F-FDG PET radiomics and clinical data using stacking ensemble learning could enhance risk stratification in DLBC [82]. The study found that radiomics features like high-intensity run emphasis (HIR) and run-length nonuniformity (RLNU) from PET and CT are independent predictors of treatment response in HL patients. Additionally, zone-size nonuniformity (ZSNU), intensity nonuniformity (INU), and short run emphasis (SRE) were identified as independent predictors of survival [83]. It has been shown that machine learning techniques using an iterated cross-validation methodology improve the predictive value for determining PFS and OS in DLBCL patients [59,84].

A multi-center study on 240 DLBCL patients using a stacking ensemble learning with ^18^F-FDG PET radiomics in combination with clinical data demonstrated superior performance in predicting PFS (AUC: 0.771, accuracy: 0.789) and OS (AUC: 0.725, accuracy: 0.763), offering better risk stratification than clinical factors alone and the International Prognostic Index [82].

Additionally, a study evaluated the predictive value of PET-based radiomics for CAR T-cell therapy outcomes in relapsed or refractory large B-cell lymphoma (LBCL). The authors reported that the radiomics signature had an AUC of 0.73, outperforming traditional biomarkers such as total TMTV with an AUC of 0.66 and SUV_max_ with an AUC of 0.59. Higher radiomic scores were linked to longer PFS and OS [85].

Combining PET texture features, specifically histogram-based features (HISTO_EntropyPET), with the German Hodgkin Group (GHSG) clinical stage effectively predicted resistance to first-line therapy in Hodgkin Lymphoma with mediastinal bulk. This approach achieved over 70% accuracy and produced distinct progression-free survival curves [86].

In relapsed or refractory (R/R) DLBCL patients undergoing CAR-T cell therapy, the combined radiomics and clinical model outperformed the clinical model alone, with AUCs of 0.776 vs. 0.712 for PFS and 0.828 vs. 0.728 for OS in the training cohort, and 0.886 vs. 0.635 for PFS and 0.778 vs. 0.705 for OS in the validation cohort [87]. The combined model integrating radiomics, clinical variables, and conventional PET parameters showed superior performance in predicting outcomes for DLBCL with extranodal involvement, achieving C-indices of 0.724 for PFS and 0.842 for OS [88].

For extranodal nasal-type NK/T cell lymphoma (ENKTCL), the combined clinical + metabolic + radiomics model showed superior predictive performance with Harrell’s C-index of 0.805 for PFS and 0.833 for OS in the validation cohort, outperforming other models [89]. In patients with DLBCL, baseline PET/CT features describing tumor spread and dissemination in relation to the spleen strongly predicted survival. Combining these features with TMTV and IPI enhanced the prediction of survival even more [90].

Machine learning models, particularly those combining MTV and MTVrate (quotient of the largest lesion’s volume and total body MTV), provided the most accurate prediction of progression-free survival in DLBCL, surpassing individual assessments [91]. The combined model incorporating RadScore, sex, B symptoms, and SUV_max_ significantly improved the prediction of medium-term efficacy and prognosis in high-risk DLBCL patients compared to individual assessments [92]. Compared to SUV-based features, ^18^F-FDG PET/CT radiomic models provided better screening results and high accuracy for T-cell lymphoma in children [93].

Draye-Carbonnier et al. [94] found that combining TMTV, tumor volume surface ratio (TVSR), and median distance between tumors (medPCD) from baseline ^18^F-FDG PET/CT improved prognosis prediction in high-burden follicular lymphoma. Moreover, integrating clinical and PET-derived features significantly improved the prediction of PFS and OS in DLBCL patients, aiding in early risk identification and personalized treatment strategies [95].

The study by Bodet-Milin et al. found that radiomic features from baseline ^18^F-FDG PET/CT, particularly from the lesion with the highest uptake, had better prognostic value for predicting minimal residual disease (MRD) in mantle cell lymphoma (MCL) patients than clinical and laboratory parameters [96]. In contrast, the GAINED study indicated that adding PET and radiomic features did not improve predictions of survival and disease progression beyond clinical and treatment features alone. While clinical features achieved a mean AUC of 0.72 ± 0.06 for 2-year PFS, and PET and radiomic features had lower AUCs of 0.60 ± 0.07 and 0.62 ± 0.07, respectively. This suggests that PET and radiomic features may not add significant value, underscoring the need for further research to optimize their integration into clinical prediction models [97].

Due to the limited reliability of tumor size in predicting treatment response in lymphoma, exploring alternative prognostic markers like radiomics is advisable. However, small sample sizes may affect study findings [3]. While these predictive models could be valuable, further research is needed. Future studies should focus on larger, diverse datasets and emphasize external validation with multi-institutional datasets and different image reconstruction algorithms. Additionally, the retrospective bias in past studies highlights the need for more prospective research.

### 4.2. Connecting Genes and Images: Radiogenomics Perspective

Radiogenomics, a promising field in oncology, holds significant potential in precision medicine. It integrates extensive radiomic features extracted from medical images, genetic information obtained through high-throughput sequencing, and clinical–epidemiological data into mathematical models. This fusion of radiomics and genomics offers an avenue to enhance our understanding of the molecular mechanisms underlying tumor development. Furthermore, it provides valuable insights to identify unique traits in cancer patients, facilitate clinical decision-making by predicting prognosis, and advance the creation of personalized treatment strategies [4].

Despite the importance of combining radiomics and genomics, there has been limited research in the context of lymphoma. In this section, we review these studies and highlight potential avenues for future research in this area. In the following section, we discuss the application of radiogenomics in the management of lymphoma.

In an effort to establish a strong connection between chemoresistance and altered metabolism in DLBCL, researchers successfully identified a 6-gene metabolic signature (T-GEP). A predictive Rad-Sig that was produced by combining FDG-PET radiomics and T-GEP data was significantly correlated with metabolic gene expression profiling (GEP)-based signature (r = 0.43, *p*-value = 0.0027), and it was also associated with PFS (*p*-value = 0.028) [98]. This highlights FDG-PET radiomics’ potential as a noninvasive tool for determining cancer metabolism and improving prognostic precision in DLBCL patients. Additionally, combining imaging, clinical, and genomic information successfully predicted treatment response in DLBCL patients, with BCL6 amplification and particular radiomic features serving as important determinants [99].

The study led by Patricio et al. employs advanced ML algorithms to analyze gene expression profiles, aiming to predict treatment responses in CHL patients after two chemotherapy cycles. Through the use of feature selection techniques, they successfully identified 14 genes (such as CD9, CD79B, BCL10, and others) strongly associated with post-chemotherapy FDG-PET results [100]. In summary, these results signify improved treatment response prediction in HL disease while emphasizing the ongoing need for further advancements in clinical applicability.

A study with a small number of B-cell lymphoma patients reported that the combined use of ^18^F-FDG PET/CT radiomics and genomic factors provided added value in predicting the survival prospects of BCL patients receiving CAR T-cell treatment [101]. However, it is worth noting that no significant association was observed between PET/CT variables and the occurrence of cytokine release syndrome. Imaging biomarkers, such as SUV_max_ derived from FDG, exhibited a moderate and approximately equal correlation with the Proliferation Index Ki-67 in lymphomas [102]. In addition, significant correlations were found between blood-based biomarkers and PET features in 30 cHL patients, specifically MTV and dissemination [103]. Future research should investigate combining blood-based biomarkers with FDG-PET radiomics to improve treatment response and prognosis prediction in cHL and other lymphoma subtypes. It is recommended to further investigate the relationship between radiomic features and the Ki-67 Proliferation Index.

As discussed in Section 4.1, promising research focused on predicting treatment response and survival using radiomics. However, there have been limited studies exploring the potential integration of radiomics with genomic information.

### 4.3. Future Prospects in Genomics and Radiomics: Liquid Biopsy and Radiomics in Lymphoma

FDG-PET is a crucial component in the management of malignant lymphomas [6]. Meanwhile, circulating tumor DNA (ctDNA), an emerging biomarker, provides genotypic insights and evaluates treatment effectiveness by detecting minimal residual disease (MRD) in lymphomas [104]. Quantitative, mutational, and fragmentation features in ctDNA dynamically predict treatment response and survival, capturing the complete lymphoma ecosystem that extends beyond traditional approaches [105]. Combining LiqBio-MRD with PET/CT results in strong diagnostic accuracy for identifying FL patients with rapid progression, underscoring LiqBio-MRD’s value in early high-risk patient identification and precision therapy [106].

Radiomics and liquid biopsy offer significant promise in oncology due to their minimally invasive nature, ease of execution, and potential for repeated assessments during patient follow-ups. These methods allow for the extraction of valuable insights into tumor type, aggressiveness, progression, and treatment response [107]. Recognizing the importance of liquid biopsy, it is essential to emphasize the need for further research in cancer diagnosis and treatment. Future research should prioritize exploring the correlation between radiomic features and ctDNA, potentially offering a comprehensive understanding of lymphoma progression and treatment response, ultimately benefiting patient care and outcomes.

**Table 1:** Summary of radiomics and AI-based radiomics studies conducted on lymphoma using ^18^F-FDG-PET/CT images. All studies included in this Table are retrospective, except Ceriani et al. [63], which is prospective in its validation. This distinction highlights one of the key limitations of the research landscape, as retrospective studies may introduce biases that can affect the interpretation of the findings.

Study	Year	P.N	Task	Study Design	Feature Extraction Software	Type	Classifier	Results (Based on Best Model)	Key Findings
Aide et al. [27]	2018	82	BMI Diagnosis, OS, PFS	Retrospective	LifeX (version 2.0)	DLBCL	Statistical analysis	Sensitivity 81.8%, specificity 81.7%	SkewnessH was notable feature for identifying BMI and only independent predictor for PFS
Mayerhoefer et al. [29]	2020	97	BMI Diagnosis	Retrospective	Beth Israel PET/CT viewer	MCL	ML	Rad-Sig (SUV and GLCM features): AUC = 0.73 Rad-Sig with laboratory data: AUC = 0.81	Combining radiomics texture features and laboratory data enhances BMI prediction in MLC.
Faudemer et al. [26]	2021	66	BMI Diagnosis	Retrospective	LifeX (version 5.1)	FL	Statistical analysis	Sensitivity: 70.0%, Specificity: 83.3%, PPV 77.8%, NPV: 76.9%, AUC: 0.822	Skeletal textural shows potential promise for the diagnosis BMI. Predictive features: variance (GLCM), correlation (GLCM), joint entropy (GLCM) and busyness (NGLDM)
Sadik et al. [30]	2021	153	BMI Diagnosis	Retrospective	-	HL	DL and statistical analysis	Agreement between AI method and physicians:81%	An AI-driven approach effectively identifies BMU in HL.
Kenawy et al. [25]	2020	44	BMI Diagnosis	Retrospective	CGITA toolbox (Math Works Inc., Natick, MA, USA, version 2015a)	Unspecified subtype	Statistical analysis	HILRE, HILZE, LRE, LZE, max spectrum, busyness, and code similarity: AUC > 0.682, *p* < 0.05 Univariate analysis: significant predictors of BMI were code similarity and LRE: *p* = 0.039, *p* = 0.02 respectively. Multivariate analysis: significant predictor of BMI was LRE: *p* = 0.031	BMI diagnosis is improved by the combination of ^18^F-FDG PET/CT radiomic features (particularly LRE) and visual evaluation.
Sadik et al. [31]	2022	48	BMI Diagnosis	Retrospective	-	HL	DL	Correlation between physicians’ decisions (using AI vs. not using it): Mean Kappa = 0.51 (0.25–0.80) vs. 0.61 (0.19–0.94)	An AI-based model significantly enhances consensus among physicians from various hospitals by highlighting suspicious focal BMUs in HL.
de Jesus et al. [36]	2022	120	Subtypes Differentiation	Retrospective	PyRadiomics (version 3.0)	FL and DLBCL	ML	AUC: 0.86, accuracy: 80%	ML analysis of radiomic features has the potential to offer diagnostic value in differentiating FL from DLBCL tumor lesions, surpassing the diagnostic capabilities of SUV_max_ alone.
Lovinfosse et al. [37]	2022	251	Subtypes Differentiation	Retrospective	RadiomiX Toolbox	DLBCL and HL	ML	Lesion-based approach using TLR radiomics: AUC = 0.95 patient-based approach (utilizing original radiomics and age): AUC = 0.86	Differentiating HL from DLBCL tumors can be achieved using ML and radiomic features.
Yang et al. [38]	2023	165	Differentiation lymph node metastasis vs. lymphoma involvement.	Retrospective	-	Lymph node metastasis and lymphoma involvement	DL	AUC = 0.901, accuracy = 86.96%, sensitivity = 76.09%, specificity = 94.20%	DL-based CAD systems exhibit promising diagnostic capabilities in distinguishing between metastatic and lymphomatous involvement in enlarged cervical lymph nodes.
Frood et al. [56]	2022	229	2-EFS	Retrospective	PyRadiomics (version 2.2.0)	DLBCL	ML	Combine model (radiomic features and clinical): AUC (validation vs. test) = 0.75 vs. 0.73	Integrating clinical characteristics with radiomic features exhibits potential in predicting 2-EFS outcomes.
Sollini et al. [58]	2020	R/R:85 Non-R/R:76	Classifying/R vs. non-R/R	Retrospective	LifeX (version 4.9)	HL	Statistical analysis	Considering all lesions vs. one single lesion: Accuracy: 82% vs. 62%, Sensitivity: 70% vs. 78%, Specificity: 88% vs. 53%	The information obtained from various lesions contributes more to predicting patient outcomes compared to relying solely on the largest lesion.
Lue et al. [60]	2020	83	PFS, OS	Retrospective	OsiriX	DLBCL	Statistical analysis	PFS (RLN_GLRLM_): HR = 15.7, *p* = 0.007 OS (RLN_GLRLM_): HR = 8.64, *p* = 0.040	RLN_GLRLM_ serves as an independent radiomics feature for predicting survival outcomes.
Parvez et al. [62]	2018	82	Response to Therapy, DFS, OS	Retrospective	LifeX	aggressive B-cell lymphoma	Statistical analysis	DFS(GLNU): *p* = 0.013, OS (kurtosis): *p* = 0.035 first-line therapy response: not significant	There is a significant correlation between GLNU and DFS, while kurtosis is correlated with OS, tumor texture features could not predict response to Therapy.
Ceriani et al. [63]	2022	133	PFS, OS	Retrospective (in validation) Prospective (in test)	PyRadiomics	DLBCL	Statistical analysis	PFS (training vs. validation): AUC: 0.709 vs. 0.706 OS: AUC: 0.740 vs. 0.703 CSS: AUC: 0.721 vs. 0.763	Rad-Sig has the potential to improve risk stratification
Li et al. [64]	2022	129	PFS	Retrospective	LifeX (version 6.10)	DLBCL	Statistical analysis	AUCs for PFS (training vs. validation set): 1 year: 0.79 vs. 0.67, 2 years: 0.84 vs. 0.83, 5years: 0.88 vs. 0.72	Combining the Rad-Score with clinical characteristics can be used to predict the outcome.
Yuan et al. [65]	2022	249	PTF	Retrospective	-	DLBCL	DL	Primary dataset: accuracy: 91.22%, AUC: 0.926 External dataset: accuracy: 88.64%, AUC: 0.925	The DL-based model demonstrated robust performance, validated the prognostic significance of interim PET/CT scans, and offers potential for informing personalized treatments.
Coskun et al. [66]	2021	45	Response to Therapy	Retrospective	LifeX (version 5.10)	DLBCL	ML and statistical analysis	Accuracy: 87%AUC = 81% GLCM dissimilarity: *p* = 0.001	Greater baseline PET image textural heterogeneity was linked to incomplete treatment response. GLCM dissimilarity were independent predictor for incomplete response
Cui et al. [67]	2023	271	2Y-TTP, PFS	Retrospective	Pyradiomic	DLBCL	ML, statistical analysis	2Y-TTP: AUC (training vs. validation vs. test) = 1.00 vs 0.83 vs. 0.898 Sensitivity (training vs. validation vs. test): 100% vs. 80.5% vs. 94.1% Specificity (training vs. validation vs. test): 100% vs. 79.2 vs. 78.1 PFS: C-index: 0.853	Integrating clinical data along with baseline, EoT, and delta PET features improve prognosis for progression/relapse after first-line therapy.
Ritter et al. [68]	2022	85	2-year EFS	Retrospective	InterView FUSION (version 3.10)	DLBCL	ML, statistical analysis	Center 1: sensitivity = 79%, specificity = 83%, PPV = 69%, NPV = 89%, center 2 (evaluating) = 0.85 AUC	Predicting 2-year EFS through radiomics and image features (Dmax, neighbor gray tone difference matrix (NGTDM) busyness, TLG, TMTV, and NGTDM coarseness) is achievable.
Jiang et al. [69]	2022	283	OS, PFS	Retrospective	PyRadiomics	DLBCL	Statistical analysis	PFS: C-index of 0.801 OS: C-index of 0.807 External validation: PFS:C-index of 0.758 OS: C-index of 0.794	Combining Rad-Sig clinical factors, it could enable precise risk stratification.
Jiang et al. [70]	2022	140	OS, PFS	Retrospective	Pyradiomics	PGI-DLBCL	Statistical analysis	Combine model (training vs. validation): PFS: (0.825 vs. 0.831), OS: (0.834 vs. 0.877)	Combining radiomics, clinical, and metabolic parameters into a unified predictive model improve the accuracy of predicting both PFS and OS.
Eertink et al. [71]	2022	317	Response to Therapy	Retrospective	RaCat	DLBCL	Statistical analysis	Combination of radiomics and clinical features: AUC 0.79 progression at 2-year TTP.: HR = 4.6	Adding radiomics to clinical predictors improved high-risk patient selection (2-year TTP progression: 44% vs. 28%), increasing PPV by 15% compared to the IPI model.
Eertink et al. [73]	2022	296	predicting 2-year progression.	Retrospective	RaCat	DLBCL	Statistical analysis	AUC = 0.72–0.75	Patient-level conventional PET features and dissemination features are the most valuable predictors for 2-year progression. Textural and morphological radiomic features did not contribute to improving the predictive value.
Travaini et al. [108]	2023	112	OS, PFS	Retrospective	LifeX (version 4.00)	DLBCL	Statistical analysis	PFS: HR = 3.56 (95% CI: 2.29–5.54, *p* < 0.0001), C-index of 0.84 (95% CI: 0.77–0.91) OS: HR = 3.83 (95% CI: 2.37–6.20, *p* < 0.0001), C-index of 0.90 (95% CI: 0.81–0.98).	Radiomic features, especially when integrated with clinical factors, demonstrated strong predictive capabilities for both PFS and OS in DLBCL patients.
Milgrom et al. [74]	2019	251	Predict Refractory Mediastinal HL	Retrospective	Imaging Biomarker Explorer (based on version 8.1.0, MathWorks)	HL	ML	AUC = 95.2%	The model utilizing PET data includes both first-order and second-order radiomic features, outperforms MTV and TLG alone, offering improved risk stratification.
Frood et al. [77]	2022	289	2-year EFS	Retrospective	PyRadiomics	cHL	ML	Training, validation and test: AUCs = 0.82, 0.79 and 0.81, respectively	It is feasible to predict outcomes using radiomic features integrated with ML model.
Jimenez et al. [78]	2022	169	Response to Therapy	Retrospective	3D Slicer software (PyRadiomics extention)	Unspecified lymphoma subtype	Statistical analysis	Response to ibrutinib treatment: AUC: 0.860, Sensitivity: 92.9%, Specificity: 81.4% at the patient level: AUC = 0.811. validated in treatment subgroups: first line: AUC = 0.916 s or greater line: AUC = 0.842 single treatment: AUC = 0.931 Multiple treatments: AUC = 0.824	The radiomics model demonstrated superior predictive accuracy for response to ibrutinib therapy.
Kostakoglu et al. [79]	2022	1263	PFS and OS	Retrospective	PORTS radiomics toolkit	DLBCL	Statistical analysis	All patients (N = 1263): PFS: AUC = 0.74, OS: AUC = 0.92 Survival probabilities at 2 years (low, intermediate, high risk) PFS: 94%, 72%, 54% OS: 100%, 100%, 51 COO subgroup analysis (n = 832): PFS: AUC = 0.86, OS: AUC = 0.89 PFS: 88%, 86%, 45% OS: 91%, 93%, 65%	PET-based prognostic model, (incorporating radiomics, PET metrics, and clinical factors) outperforms the traditional IPI in predicting survival
Zhao et al. [82]	2023	240	PFS and OS	Retrospective	LifeX (version 6.3)	DLBCL	ML	PFS: AUC = 0.771, accuracy = 0.789. OS: AUC = 0.725 and an accuracy of 0.763.	The combined model using ^18^F FDG PET radiomics and clinical data improves risk stratification in DLBCL patients.
Wang et al. [109]	2020	110	PFS and OS	Retrospective	LifeX (version 5.1)	ENKTL	Statistical analysis	PFS (training vs. validation): Radiomics-based Model: C-index = 0.811 vs. 0.588 Metabolism-based Model: C-index = 0.751 vs. 0.693 OS (training vs. validation): Radiomics-based Model: C-index = 0.818 vs. 0.628 Metabolism-based Model: C-index = 0.828 vs. 0.753. R-signature: PFS: HR = 3.56 (95% CI: 2.29–5.54, *p* = 0.001), C-index of 0.84 (95% CI: 0.77–0.91) OS: HR = 3.83 (95% CI: 2.37–6.20, *p* = 0.032), C-index of 0.90 (95% CI: 0.81–0.98).	Combining Rad-Sig and clinical factors demonstrate robust predictive capabilities. In the validation phase, the radiomics-based model demonstrated lower effectiveness compared to the metabolism-based models.
Lue et al. [83]	2020	35	Response to Therapy, OS, PFS	Retrospective	CGITA (MATLAB 2012a)	HL	Statistical analysis	Treatment Response Prediction: HIR_GLRM_PET_: OR = 36.4, *p* = 0.014 RLNU_GLRM_CT_: OR = 30.4, *p* = 0.020 PFS: INU_GLRM_PET_: HR = 9.29, *p* = 0.023 Wavelet SRE_GLRM_CT_: HR = 18.40, *p* = 0.012 OS: ZSNU_GLSZM_PET_: HR = 41.02, *p* = 0.001 A prognostic stratification model: PFS (*p* < 0.001), OS (*p* < 0.001).	Predictive features for treatment response HIR_GLRMPET and RLNU_GLRMCT. Independent predictive features for survival: ZSNU(GLSZM_PET_), INU(GLRM_PET_), and wavelet SRE (GLRM_CT_)
Chang et al. [84]	2023	122	PFS, OS	Retrospective	-	DLBCL	ML and DL	Best Predictive Models for PFS: accuracy 0.71, Sensitivity: 0.58, Specificity: 0.84, AUC: 0.71, PPV: 0.75, NPV: 0.71, F1 score: 0.62 Best Predictive Model for OS: accuracy of 0.76, Sensitivity: 0.59, Specificity: 0.84, AUC: 0.72, PPV: 0.75, NPV: 0.75, F1 score: 0.66	ML methods, utilizing clinical and metabolic features, improve PFS, OS prediction.
Zhang et al. [61]	2021	152	PFS, OS	Retrospective	LifeX (version 6.30)	DLBCL	Statistical analysis	PFS (training vs. validation) (all *p* < 0.05): AUCs of TMTV-based hybrid nomogram (combining RS with IPI) 0.828 vs. 0.783 AUCs of MBV-based hybrid nomogram: training 0.835 vs. 0.787 OS (all *p* < 0.05): AUCs of TMTV-based hybrid nomogram: 0.818 vs. 0.789 AUCs of MBV-based hybrid nomogram: 0.831 vs. 0.792	The hybrid nomograms utilizing Rad-Sig with IPI could significantly improve survival prediction.
Mazzara et al. [98]	2023	112	PFS	Retrospective	LifeX (version 5.1)	DLBCL	Statistical analysis	Rad-Sig ((histo kurtosis, histo energy, shape sphericity, and neighboring gray level dependence matrix contrast), significantly associated with the metabolic GEP–based signature (r = 0.43, *p* = 0.0027) PFS: *p* = 0.028	A mitochondrial metabolism-related gene expression pattern correlates with specific radiomic features and patient outcomes. Combining FDG-PET radiomics with MTV enhances the accuracy of PET-based prognosis.
Ferrer-Lores et al. [99]	2023	33	Response to Therapy	Retrospective	Texture analysis Quibim	DLBCL	Statistical analysis	Combine model: AUC = 0.904, accuracy = 90% Predictive model (manual): GLSZM_GrayLevel, Variance: *p* = 0.048, Sphericity: *p* = 0.027, GLCM_Correlation: *p* = 0.05, BCL6 amplification = *p* = 0.018	A successful prediction of the response to initial treatment was achieved by combining imaging characteristics, clinical factors, and genomic data. GLSZM (GrayLevelVariance), Sphericity and GLCM(Correlation) were predictors of response. The amplification of BCL6 emerged as a highly predictive genetic marker.
Zhou et al. [101]	2022	24	predict survival outcomes (using radiomics and genomics data)	Retrospective	LifeX (version 6.30)	B-Cell	Statistical analysis	PFS: NGLDM: HR = 15.16, *p* = 0.023 MYC and BCL2 double-expressor (DE): HR = 7.02, *p* = 0.047 The integration of NGLDM_Contrast_PET_ and DE: Group 1 (risk factors = 0; Number of patients: 7): PFS: 85.7% and OS: 100% Group 2 (risk factor = 1; Number of patients: 11): PFS: 63.6% and OS: 90.9% Group3 (risk factors = 3; Number of patients: 6): PFS: 0% and OS: 16.7%	Combining ^18^F-FDG PET/CT radiomic features with genomic factors has the potential to forecast the survival outcomes of B-Cell patients undergoing CAR T-cell therapy. there was no noteworthy correlation found between PET/CT variables and CRS.
Triumbari et al. [110]	2023	227	PFS, DS	Retrospective	Moddicom	cHL	Statistical analysis	PFS (mainly depended on hottest lesion): AUC: 0.74 DS (mainly depended on largest lesion): AUC: 0.78	Radiomics models that focus on the largest and most active lesions offer valuable insights into the prognosis of patients.
Zhou, et al. [87]	2023	61	PFS, OS	Retrospective	LifeX (version 6.30)	DLBCL	Statistical analysis	PFS: C-Index = 0.710 vs. 0.640 in validation, AUC = 0.776 vs. 0.886 OS: C-Index = 0.780 vs. 0.676, AUC = 0.828 vs. 0.778	The radiomics model consistently outperforms in predicting both PFS and OS, not only in the main analysis but also during validation, highlighting its superior performance.
Albano, et al. [111]	2024	137	Detecting Richter transformation (RT), OS	Retrospective	LifeX (version 6.30)	Chronic lymphocytic leukemia (CLL)	Statistical analysis	Richter Transformation (RT): In 130 out of 137 (95%) PET/CT scans, increased tracer uptake was observed. SUVbw, SUVlbm, SUVbsa, L-L SUV ratio, and L-BP SUV ratio were significantly higher in the RT group (*p* < 0.001). OS: Median OS was 28 months for patients with RT and 34 months for those without RT (*p* = 0.002).	Higher SUV metrics and L-BP SUV ratio were linked to worse outcomes in RT, with a median OS of 28 months versus 34 months without RT.
Yousefirizi, et al. [112]	2024	31	Relapse/Progression and TTP Prediction	Retrospective	Pyradiomics	Primary mediastinal large B-cell lymphoma	ML	Relapse/Progression (Delta radiomics): Accuracy: 0.89 ± 0.03 F1 Score: 0.89 ± 0.03 Best Model (TTP): C-index = 0.68 ± 0.09 (EoT radiomics)	Delta radiomics significantly improved the prediction of relapse/progression compared to EoT radiomics. Combining baseline and Delta radiomic features was as effective as using EoT radiomics alone for predicting TTP in PMBCL patients.
Travaini et al. [108]	2024	112	PFS, OS	Retrospective	LifeX (version 6.32)	DLBCL	Statistical Anlysis	PFS: C-Index = 0.84 (0.77–0.91) OS: C-Index = 0.90 (0.81–0.98)	The combined clinical–radiomic model is better for predicting PFS, OS in DLBCL patients compared to using radiomics or clinical parameters alone.

Progression-free survival: PFS, overall survival: OS, negative predictive value: NPV, positive predictive value: PPV, 2-year event-free survival: 2-EFS, hazard ratio: HR, radiomics Score: RS, primary treatment failure: PTF, 2-year time to progression: 2Y-TTP, gene expression profiling: GEP, cytokine release syndrome: CRS, run length non-uniformity extracted from a gray level run length matrix: RLNGLRLM, gray-level non-uniformity: GLNU, area under the curve: AUC, computer-aided diagnosis: CAD, standardized uptake value: SUV, Bone marrow involvement: BMI, mantle cell lymphoma: MCL, follicular lymphoma: FL, diffuse large b cell lymphoma: DLBCL, bone marrow uptake: BMU machine learning, deep learning, radiomics signature: Rad-Sig, gray-level co-occurrence matrix: GLCM, neighboring gray-level dependence matrix: NGLDM, End of Therapy: EoT, cell-of-origin: COO, High-intensity run emphasis: HIR, run-length nonuniformity: RLNU, gray-level run-length matrix: GLRM, intensity nonuniformity: INU, wavelet short run emphasis: SRE, zone-size nonuniformity: ZSNU, gray-level size zone matrix: GLSZM, odd ratio: OR, total metabolic tumor volume: TMTV, metabolic bulk volume: MBV, extranodal natural killer (NK)/T cell lymphoma: ENKTL, classical hodgkin lymphomas: cHL, international prognostic index: IPI, tumor-to-liver ratio: TLR, Deauville score at interim PET/CT: DS, refractory/relapse: R/R, concordance index: C-index, patients’ number: P.N, primary gastrointestinal-DLBCL: pGI-DLBCL, cause-specific survival: CSS, the maximum standardized uptake value body weight: SUVbw, lean body mass: SUVlbm, body surface area: SUVbsa, lesion-to-blood-pool SUV ratio: L-BP SUV R, lesion-to-liver SUV ratio: L-L SUV R.

### 4.4. AI-Driven Lymphoma Diagnosis and Prognosis Tool: Synergizing AI and PET/CT

These days, PET/CT imaging is essential to the treatment of lymphoma patients [5]. However, because affected nodes, organs, and physiological uptake patterns vary, identifying and accurately demarcating lymphoma lesions on whole-body FDG-PET/CT scans presents considerable challenges [113]. Accurate segmentation in PET/CT images is vital for radiotherapy treatment planning [114] and prognostic evaluation. Parameters, such as Total MTV (TMTV) and total lesion glycolysis (TLG), extracted from segmented images, serve as valuable prognostic markers [115,116,117]. Unfortunately, due to the limitations of data in this type of imaging, performing this task manually has several drawbacks. The inherent challenges of this imaging technique make it extremely time-consuming and susceptible to human error [69]. Artificial intelligence (AI) is developing quickly and has a lot of potential to help physicians diagnose and treat cancer patients [118]. We will explore the possibility of combining AI and PET/CT for better lymphoma treatment in the next section.

#### 4.4.1. Imaging-Based Biomarkers Prediction

**Figure 7:**
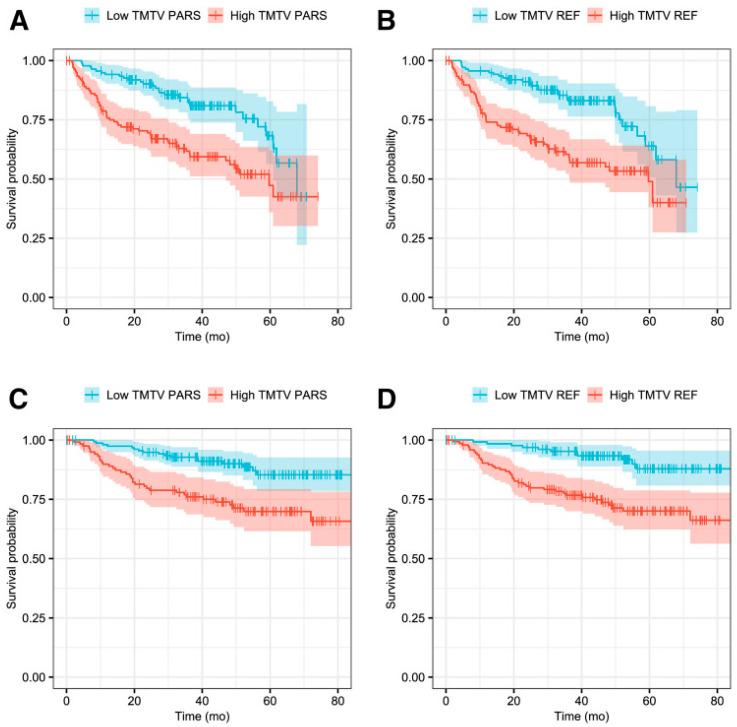
Kaplan–Meier survival plots representing PFS in panels (**A**,**B**), and OS in panels (**C**,**D**). Reprinted with permission from [119] under a Creative Commons Attribution 4.0 International License.

In recent years, MTV has proven to be a valuable prognostic imaging-based biomarker in lymphoma. Additionally, in DLBCL patients, TMTV demonstrated notable prognostic significance in predicting both PFS and OS (Figure 7) [119,120,121]. However, current manual methods for measuring MTV are time-consuming, emphasizing the necessity of developing an automated MTV calculation method with the assistance of deep learning [122,123]. In a recent study, it was found that AI-driven software for detecting lesions provides automated, rapid, dependable, and consistently effective solutions for regularly acquiring measurements of TMTV and TLG [124]. Measuring D_max_, the largest distance between lymphoma sites, as a robust independent prognostic factor in DLBCL patients highlights its potential clinical significance.

**Figure 8:**
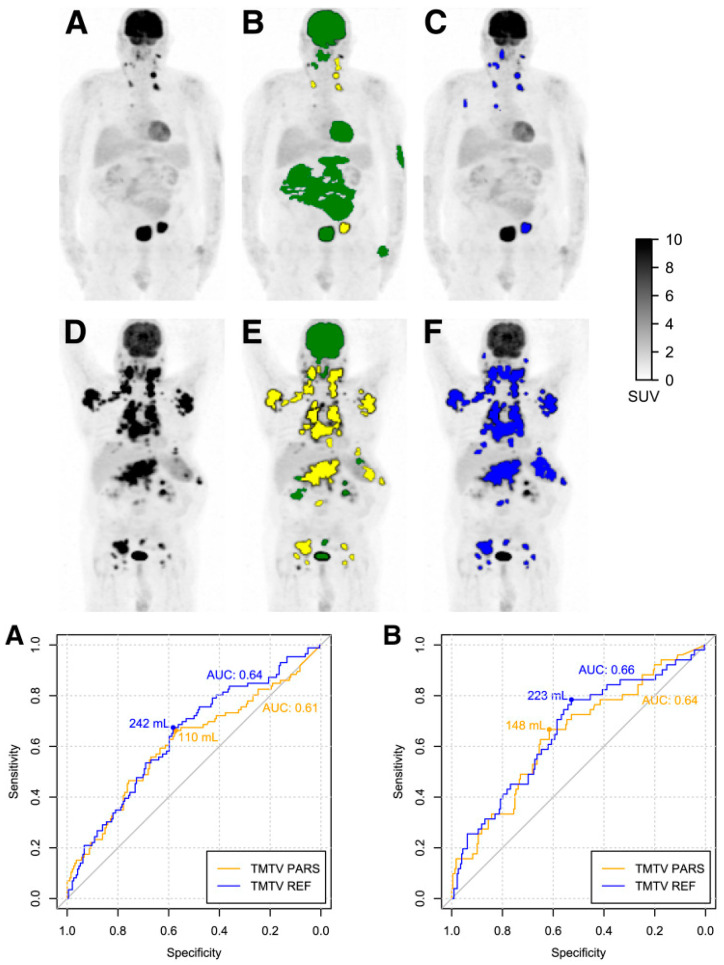
(**Top**) panel: Identification and categorization of regions with elevated ^18^F-FDG absorption as either physiological or anomalous. (**A**,**D**) Maximum-intensity-projection PET visuals of subjects with low TMTV (**A**) and high TMTV (**D**). (**B**,**E**) ROI_PARS_ generated automatically via the PARS software prototype. ROI_PARS_ regions pinpointed by the MFS algorithm are superimposed on the PET maximum-intensity projection. Physiologically classified ROI_PARS_ sites by the deep-learning mechanism are depicted in green, while those deemed suspicious are in yellow. (**C**,**F**) ROI_REF_ delineated by a seasoned nuclear medicine expert utilizing semi-automatic tools. (**Bottom**) panel: Receiver-operating-characteristic curves for TMTV_PARS_ and TMTV_REF_ pertaining to 4-year PFS (**A**) and 4-year OS (**B**). Corresponding AUC values and ideal TMTV thresholds are indicated. Modified and reprinted from [119] under a Creative Commons Attribution 4.0 International License.

This research raises the possibility of using this measure in deep learning-guided segmentation techniques for clinical evaluation [125]. Prognostic biomarkers, such as TMTV [126,127,128], Dmax [127], and MTV [122,129], can be automatically segmented and estimated in DLBCL patients by AI-based algorithms, more specifically, CNN-based algorithms. For DLBCL patients, these techniques can produce quantitative data in the form of a predictive TMTV for prognosis prediction [129]. Figure 8 shows a comparison between the performance of a human reader and CNN-based automatic TMTV calculation.

In comparison with conventional deep learning (CDL), weakly supervised deep learning (WSDL) demonstrated higher effectiveness in extracting ^18^F-FDG PET/CT features and predicting the prognosis in ENKTL patients. This superiority is attributed to the utilization of a larger training dataset and the capability to refine unclean data. WSDL and CDL achieved notable prediction sensitivities of 87.50% and 62.50%, specificities of 83.33% and 83.33%, and accuracies of 85.00% and 75.00%, respectively [81]. To establish it as a robust clinical tool, potentially providing confidence levels for risk classification, future research should focus on validating this CNN-based approach in larger multicenter cohorts.

Jiang et al. conducted a study using deep learning to develop PET-based imaging biomarkers for precise survival prediction in DLBCL patients. The results demonstrated the efficacy of these biomarkers in predicting both PFS and OS. Furthermore, multiparametric models that incorporated these biomarkers surpassed other models in both the training (PFS: C-index = 0.866 and OS: C-index = 0.835) and external validation cohorts (PFS: C-index = 0.760 and OS: C-index = 0.770), underscoring their potential for stratifying patient risk [130].

Lymphoma segmentation is crucial in clinical diagnosis because it involves accurate identification and the outlining of lymphoma lesions in medical images, such as ^18^F-FDG PET/CT scans. This precision is essential for determining the extent of the disease, planning effective treatments, monitoring how tumors respond to therapy, and extracting detailed radiomic features that aid in prognosis. Effective segmentation ensures that clinicians have the necessary information to make informed decisions, leading to more personalized and successful patient care. It is especially important for distinguishing between malignant and benign lymphomas and is essential for treatment planning. In this context, several studies have developed innovative DL models to enhance the precision and reliability of delineating lymphoma boundaries in PET images (Dice coefficients of 87.18% and 0.8796) [131,132]. Similarly, a different study presented another proposed deep learning-based semi-automated lymphoma segmentation approach (Dice coefficient = 0.907) [133]. Additionally, a separate study introduced a computer-aided diagnosis (CAD) system to accurately segment ENKT lymphoma lesions (Dice coefficient = 0.7115, sensitivity = 0.7472) [134]. A recent study has introduced an unsupervised image generation approach that leverages anatomical-metabolic consistency representations. This method significantly improves the accuracy of lymphoma segmentation in PET/CT images, demonstrating substantial potential for practical clinical applications and aiding clinical diagnosis [75].

Eertink et al. reported that the utilization of various data segmentation techniques resulted in differences in radiomics feature values and the selection of specific features for DLBCL [135]. Nonetheless, disparities can be found in the actual values of radiomic features acquired and the selected features when utilizing various segmentation techniques. Significant correlations were identified among MTV, intensity, and various dissemination features derived from different segmentation techniques, indicating a persistent and reliable prognostic performance. Although a threshold-based segmentation using an SUV level of 4.0 offers practical advantages in cHL PET/CT segmentation, its effectiveness with smaller, less avid lesions requires further evaluation in a larger patient cohort to confirm its clinical applicability [136]. A multi-resolution 3D U-Net model for TMTV segmentation in lymphoma achieved an average Dice score of 0.68 on internal data and 0.66 on multi-center external data, with a correlation of 0.89 to the ground truth TMTV [137].

In the medical domain, CNN’s reliance on precise pixel-level annotations poses challenges in terms of time and expertise needed for accurate medical image analysis. To address this, researchers have introduced an innovative WSD model utilizing weak labels and multi-scale feature similarity in PET/CT images [69]. Future work should aim to enhance the performance of the algorithms dedicated to weak and/or noisy datasets, optimize computational efficiency, expand dataset diversity for validation, and investigate real-world clinical application feasibility.

#### 4.4.2. System-Aided Diagnosis and Staging

The utilization of PET/CT scans holds a pivotal position in the initial staging, determining the most appropriate therapy. It excels in identifying lymphoma involvement in areas that might be overlooked by CT scans alone. This capability is essential in avoiding the risk of undertreating patients with advanced disease stages who could otherwise be mistakenly classified as having limited-stage disease solely based on CT findings. In essence, FDG-PET/CT significantly enhances our ability to make precise treatment decisions in lymphoma cases [5].

The Ann Arbor classification continues to serve as the gold standard staging system for non-cutaneous lymphomas, categorizing lymphoma patients into four distinct disease stages. In accordance with the Lugano classification, FDG-PET/CT is now routinely recommended for all FDG-avid lymphomas, representing a professional consensus in modern lymphoma staging and diagnosis [5,138]. The Deauville 5-point scale can also be considered a treatment response evaluation system in lymphoma patients based on PET/CT images. This system can also lead to variations in assessments among different observers. To address this challenge, Sadik et al. introduced an AI-driven method to quantify reference levels in both the liver and the mediastinal blood pool [139]. In the validation group, AI-based measurements demonstrated good agreement with radiologists (in the validation group, the mean difference for quantifying reference levels was 0.02 in both the liver and the mediastinal blood pool). One key study showed that domain adaptation significantly enhanced language models’ ability to predict five-point Deauville scores from PET/CT reports [140]. Deep learning effectively differentiates ^18^F-FDG-PET-CT scans of lymphoma patients with and without hypermetabolic tumor sites, suggesting its potential as a tool for ruling out metabolically active disease or as a second reader in a clinical setting (achieving an AUC of 0.953, accuracy of 0.907, sensitivity of 0.874, and specificity of 0.949 in external testing) [141].

In DLBCL patients, deep learning scores from PET images provide valuable survival prognostication, with multiparametric models achieving C-indices of 0.866 for PFS and 0.835 for OS in training, and 0.760 and 0.770 for PFS and 0.748 and 0.766 for OS in external validation [130]. Recent research found that deep learning-enhanced PET imaging improved image quality and diagnostic accuracy while reducing imaging duration by 50% for lymphoma patients [142]. The hybrid few-shot multiple-instance learning model achieved 0.75 accuracy and 0.795 AUC in predicting lymphoma aggressiveness [143].

As mentioned at the beginning of this section, lymphoma detection can be prone to misinterpretation by physicians due to the inherent limitations of this type of imaging. The integration of artificial intelligence-driven automatic detection and segmentation algorithms has the potential to significantly enhance the efficiency and accuracy of physicians in analyzing these images [69].

In this regard, Yu et al. introduced a novel semi-automatic graphical model-based approach for both detection, achieving a 100% detection rate, and segmentation, with a Dice coefficient of 84.4% [144]. ^18^F-FDG PET/CT scans offer valuable prognostic insights for individuals with MCL [145]. However, manual detection of MCL lesions on these scans is labor-intensive and not currently integrated into routine clinical procedures. DL-based models show promising levels of accuracy in detecting MCL on PET/CT images, suggesting potential wider clinical applications. In another study, a substantial enhancement in the classification accuracy of areas showing normal physiological FDG uptake and excretion in lymphoma PET/CT scans was observed (F1-score of 91.73%). This improved precision can aid in early detection, improved treatment, and prognostic assessments for lymphoma patients, all while minimizing false positives during image interpretation [146].

**Figure 9:**
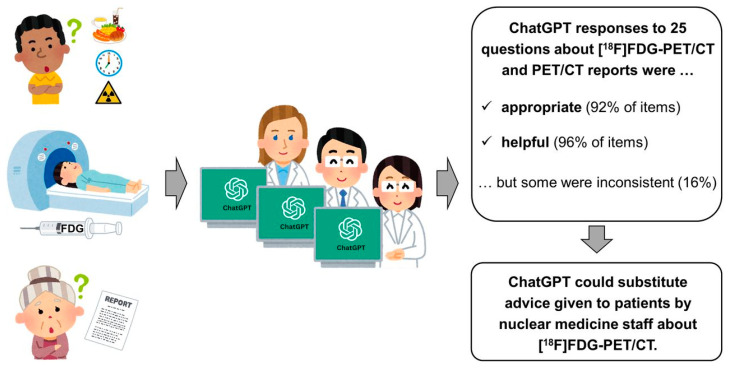
The study with thirteen questions and six PET/CT reports found ChatGPT’s responses “appropriate” 92% and “useful” 96% of the time, though 16% were inconsistent. This work was originally published in JNM [147].

Given the growing use of chatbots for everyday problem-solving, researchers recently evaluated if an AI-based language model, specifically ChatGPT, developed by OpenAI, can effectively answer patient questions about ^18^F-FDG PET/CT scans. As shown in (Figure 9), the study involved thirteen questions and six PET/CT reports (lung cancer, Hodgkin lymphoma). Nuclear medicine staff rated the responses for appropriateness and usefulness. Results showed ChatGPT provided “appropriate” responses for 92% and “useful” answers for 96% of the questions; however, 16% of responses were inconsistent [147]. 

In a recent study, Constantino et al. found that the Self-Adaptive Configuration, SAC (Bayesian method outperformed others in robustness, reproducibility, and accuracy on [^18^F]FDG PET/CT lymphoma images, achieving the highest intra-observer Dice Coefficient (0.87) and inter-observer DC (0.94), surpassed manual segmentation in tumor quantification [148]. It can replace manual segmentation for lymphoma lesions, ensuring consistent quantitative measurements and facilitating the creation of accurate, large datasets for deep learning-based segmentation models. The study advises against using absolute or relative threshold-based methods in clinical studies due to potential substantial differences in important lesion features, such as SUV_mean_, MTV, and TLG, compared to manual segmentation. For future research, it is recommended to conduct tests on a larger number of patients, explore the combination of PET and CT images to enhance unary energy using graph-based models, and develop a more comprehensive multi-atlas to reduce false positives.

**Table 2:** Summary of AI-assisted studies conducted on lymphoma using ^18^F-FDG PET/CT images. All studies in this table are retrospective, except for Blanc-Durand et al. [126], which is prospective. This difference emphasizes a key limitation in the research field, as retrospective studies may introduce biases that could impact the interpretation of the results.

Study	Year	P.N	Task	Study Design	Subtype	Algorithms	Results	Clinical Applications	Key Findings
Capobianco et al. [119]	2021	301	Automatic calculation of MTV Classification of high-uptake regions PFS, OS	Retrospective	DLBCL	CNN (classification), Survival: statistical analysis	Correlation between TMTV_PARS_ and TMTV_REF_: (ρ = 0.76; *p* < 0.001). The classification accuracy was 85%. Sensitivity was 80%. Specificity was 88%. PFS: HR for TMTV_PARS_: 2.3 (*p* < 0.001). HR for TMTV_REF_: 2.6 (*p* < 0.001). OS: HR for TMTV_PARS_: 2.8 (*p* < 0.001). HR for TMTV_REF_: 3.7 (*p* < 0.001).	Simplified TMTV estimation, reduced observer Variability, provide valuable prognostic information to improve patient care and decision-making.	DL-based automation can estimate TMTV and underscores its significance as a valuable prognostic tool. The resulting TMTV, obtained through this automated DL, demonstrated significant prognostic value for both PFS, OS.
Pinochet et al. [120]	2021	119	Automatic calculation of MTV	Retrospective	DLBCL	DCNN	median DSC score: 0.65, ICC between automatically and manually obtained TMTVs: 0.68. PFS (automatically based TMTV vs. manually based TMTV): HR = 2.1 vs. 3.3 OS: HR = 2.4 vs. 3.1	Enhance cancer detection, provide valuable prognostic information, tailor treatment plans, and support clinical decision-making	Performance and predictive value of both automatic and manual TMTV measurements for PFS and OS in the respective patient cohorts.
Huang et al. [69]	2020	147	Automatic Segmentation	Retrospective	DLBCL	WSDL	DSC = 71.47%.	Reduced dependence on expert annotations Improving the diagnosis, treatment planning, and monitoring of DLBCL	Their method accurately segmented lymphoma in PET/CT images using weak labels, reducing the need for expert annotations.
Kuker et al. [122]	2022	100	Automatic calculation of MTV	Retrospective	DLBCL	DCNN	Reader 1 vs. AM (Automated Method): Pearson’s Correlation Coefficient: 0.9814 (*p* < 0.0001) ICC: 0.98 (*p* < 0.001) Reader 2 vs. AM (Automated Method): Pearson’s Correlation Coefficient: 0.9818 (*p* < 0.0001) ICC: 0.98 (*p* < 0.0001)	Automated MTV Calculation Enhances the reliability of prognostic biomarkers, and has implications for treatment planning	The automated MTV calculation closely matches nuclear medicine reader measurements, offering efficiency and reproducibility without extensive training data, suitable for clinical research.
Karimdjee et al. [124]	2023	51	Automatic calculation of MTV	Retrospective	DLBCL	Methods 1 and 2: fully automated with exclusion of lesions ≤0.5 mL and ≤0.1 mL, respectively. Methods 3 and 4: fully automated with physician review. Method 5: semi-automated	For the main user between methods 3 and 5: ICC for TMTV: 0.99, ICC for TLG: 1.0 Between the two users applying method 3: ICC for TMTV: 0.97, ICC for TLG: 0.99 Mean processing time (±standard deviation): Method 1: 20 s ± 9.0 Method 3: 178 s ± 125.7 Method 5: 326 s ± 188.6 (*p* < 0.05)	Enhances the precision and efficiency of cancer diagnosis and treatment monitoring in a clinical workflow.	AI-based lesion detection software is a reliable tool for daily TMTV and TLG measurements.
Blanc-Durand et al. [126]		733	Automatic calculation of TMTV	Prospective	DLBCL	CNN	Validation Set Results: DSC: 0.73 ± 0.20 Mean Jaccard Coefficient: 0.68 ± 0.21 Underestimation of mean TMTV: −116 mL (20.8%) ± 425 (statistically significant, *p* = 0.01) Training (n = 693): Underestimation of mean TMTV: −12 mL (2.8%) ± 263 (not statistically significant, *p* = 0.27)	Accurate tumor volume assessment. helps oncologists determine the optimal treatment strategy. diagnostic assistance, increasing the reproducibility of TMTV assessment	CNN holds promise for automating the detection and segmentation of lymphoma lesions.
Girum et al. [127]	2022	382	Automatic calculation of MTV and D_max_ using 2MIPs	Retrospective	DLBCL	CNN	Correlation coefficients: sTMTV correlation with TMTV for center 1: Spearman r = 0.878 sTMTV correlation with TMTV for center 2: Spearman r = 0.752 sD_max_ correlation with D_max_: r = 0.709 D_max_ correlation with D_max_: r = 0.714 Hazard Ratios for PFS (95% CI): TMTV: 11.24, sTMTV: 11.81, D_max_: 9.0, sD_max_: 12.49	Uniformity in Biomarker Computation. Calculation of biomarkers. Marked Improvement in Diagnostic and Prognostic Efficiency Simplify the computation and application of these characteristics in clinical settings	AI algorithms can automatically estimate surrogate TMTV and D_max_, which are prognostic biomarkers, using only 2 PET MIP images.
Zhou et al. [145]	2021	142	Detection	Retrospective	MCL	DLCNN	Center1: sensitivity of 88% (IQR: 25%) with 15 (IQR: 12) FPs/patient center 2: sensitivity of 84% (IQR: 24%) with 14 (IQR: 10) FPs/patient	Improved diagnostic accuracy, reduced labor-intensive analysis, early detection and staging	DLCNN demonstrated high sensitivity for the detection of MLC

PET-assisted reporting system: PARS, deep convolutional neural network: DCNN, surrogate TMTV: sTMTV, surrogate D_max_: sD_max_, metabolic tumor volume: TMTV, total lesion glycolysis: TLG, intra-class coefficient: ICC, weakly supervised deep learning: WSDL, dice similarity coefficient: DSC, deep learning: DL, progression-free survival: PFS, time-to-progression: TTP, overall survival: OS, diffuse large b cell lymphoma: DLBCL, maximum tumor dissemnination: D_max_, mantle cell lymphoma: MCL, interquartile range: IQR, false positives: FPs, maximum intensity projections: MIPs, patients number: P.N.

## 5. Conclusions and Future Directions

This review provides an up-to-date overview of the utilization of PET/CT radiomic features and artificial intelligence in the management of lymphoma patients, highlighting their significant implications for precision medicine and improved patient care. However, further investigation is needed before these methods can be incorporated into routine clinical practice.

Radiomic features derived from ^18^F-FDG PET/CT scans have the potential to provide insights into bone marrow involvement (BMI) status in lymphoma patients [25,28,29,30,31,96]. Studies have shown that combining radiomic features with clinical data enhances diagnostic accuracy, with area under the curve (AUC) values ranging from 0.68 to 0.822, sensitivity up to 81.8%, and specificity up to 83.3%. These results indicate from moderate to high effectiveness in detecting BMI, surpassing traditional diagnostic methods like bone marrow biopsy (BMB). However, the limited number of studies and the lack of multi-institutional large-scale research prevent us from reaching a definitive conclusion. Utilizing a multifaceted approach that integrates clinical, biological, and PET features could improve the diagnosis of BMI in lymphoma patients. Extensive prospective clinical studies with larger sample sizes are necessary to assess the practicality of ^18^F-FDG PET/CT radiomics in a clinical setting.

While some studies have highlighted the potential of radiomic features and machine learning (ML)-based radiomics models in differentiating various tumor types [39,40,41,42,43,44,45,46,47,48], there is a limited number of studies focusing on distinguishing the histological subtypes of lymphoma [35,36,37]. Radiomics and ML models have effectively distinguished between different lymphoma subtypes, achieving AUCs as high as 0.95 and accuracies up to 86.96%, outperforming conventional metrics, such as SUV_max_ [35,36,37]. Additionally, radiomics may differentiate between lymph node metastasis and lymphoma involvement [38]. Future studies should be conducted on larger datasets, different lymphoma subtypes, and lymph node metastasis from primary lesions. There is hope that future research can position radiomics as an additional non-invasive tool to further investigate the characteristics of lymphoma, potentially reducing the need for invasive biopsies and leading to more personalized treatment strategies.

Most research utilizes radiomics or AI-based PET/CT radiomics models to predict prognosis or outcomes in various lymphoma subtypes, particularly diffuse large B-cell lymphoma (DLBCL). As seen in Table 1, a significant number of radiomics studies focus on DLBCL in outcome prediction and response to treatment [18,56,59,61,62,63,68,69,71,72,76,77,80,82,83,84,87,98,105,112,117,149,150,151]. These studies reported high accuracies of up to 90% and AUCs of up to 0.952, indicating that radiomics-based predictive models, especially when integrated with clinical data, can serve as valuable prognostic tools. The findings demonstrate that radiomic models often outperform traditional methods in diagnostic accuracy, treatment response prediction, and survival outcome forecasting. However, further research in this area is warranted to validate these findings across different lymphoma subtypes.

As discussed above, promising research has primarily focused on predicting treatment response and survival using radiomics. Despite the significance of integrating radiomics and genomics, there has been very limited research in the context of lymphoma. Radiogenomics offers a pathway to enhance our understanding of the molecular mechanisms underlying tumor development. Moreover, it provides valuable insights for identifying unique traits in cancer patients, facilitating clinical decision-making by predicting prognosis and advancing the development of personalized treatment strategies. Therefore, it is imperative that future research prioritizes the acquisition of knowledge through the integration of radiomics and genetic data to enhance lymphoma management.

Liquid biopsy (LB) and radiomics offer promising characteristics in the field of cancer research, as they are both quantifiable and non-invasive and have the potential to be repeated during patient monitoring [107]. However, there is a significant research gap in the untapped potential for synergy between liquid biopsy and radiomic features extracted from PET images. The exciting opportunity lies in combining the genetic insights from liquid biopsy with the quantitative data from PET images using radiomics. This synergy has the potential to enhance our understanding of lymphoma and can lead to more accurate diagnoses and tailored treatment strategies.

Furthermore, in this review, we have emphasized the significance of AI-based tools as valuable aids for medical practitioners. Over recent years, there has been a growing interest in utilizing AI to assist physicians in addressing challenges associated with the interpretation of PET/CT images. This emerging field tackles various image-related challenges, including low resolution, limited contrast, unclear boundaries, variations in tissue absorption, and individual differences. The application of AI models has proven essential for a wide range of tasks, including tumor diagnosis, image segmentation, post-treatment evaluation, pathology prediction, and estimation of survival rates.

In addition, AI holds great promise in enhancing the quality of ^18^F-FDG PET imaging [152], particularly in cases where structural imaging is unavailable. Despite these promising developments, several formidable hurdles must be overcome to practically implement AI-based physician assistant tools in clinical settings.

Radiomics has the potential to greatly improve lymphoma patient care by providing more accurate diagnoses, personalized treatment plans, and better outcome predictions. By establishing standards for data collection and feature extraction, AI models can become more accurate and stable, allowing clinicians to predict how patients will respond to treatments, such as chemotherapy or radiotherapy. Analyzing baseline images can identify patients at higher risk of relapse, enabling early adjustments in treatment strategies. Federated learning can further enhance AI models by training on large, diverse datasets without sharing sensitive patient data, thus improving prediction accuracy and applicability. Future clinical studies should focus on integrating AI-driven decision support tools into diagnosis and treatment, helping physicians offer more precise, personalized care while reducing the need for invasive procedures.

As highlighted by Hasani et al. [15], AI holds great potential in lymphoma PET imaging, particularly in tasks such as lesion detection, classification, segmentation, and predicting treatment outcomes. Manual delineation of hypermetabolic lesions is time-consuming, but AI can automate these tasks and improve tumor characterization. Implementing AI-based tools in clinical settings requires large, diverse datasets and multi-center validation to ensure robustness and generalizability.

For future research, advanced models, such as Vision Transformers (ViT) and Attention-based CNNs, can enhance quantitative image analysis, while a Hybrid AI–Human Collaboration Model can combine AI automation with human expertise. Federated learning also offers a practical solution for training models across institutions without sharing sensitive data. Addressing these challenges will enable more robust AI applications in lymphoma care.

As indicated in Table 1 and Table 2, the majority of studies included in this analysis (up to 95%) are retrospective in design. While retrospective studies are valuable, they can introduce certain limitations, such as selection and recall bias, which may influence the results. Therefore, these limitations should be carefully considered when interpreting the findings of this research.

In conclusion, the comparative analysis of current studies underscores the significant potential of radiomics and AI in improving lymphoma management. While many studies focused on DLBCL for outcome prediction and treatment response, there is a need for further research on other lymphoma subtypes, especially in the context of BMI diagnosis and histologic differentiation. The numerical results from these studies indicate that radiomic models often outperform traditional methods. However, the variability in study designs, lymphoma subtypes, and methodologies highlights the necessity for standardized approaches and larger, multi-center studies to validate these findings. We recognize that variations in reported performance metrics, including specificity, sensitivity, and area under the curve (AUC), pose significant challenges in directly comparing the predictive performance of different studies. By addressing these challenges, radiomics and AI can be more effectively integrated into routine clinical practice, ultimately enhancing patient care and outcomes in lymphoma.
